# Ten years after: findings from the medical surveillance program on Health Effects in High-Level Exposure to PCB (HELPcB)

**DOI:** 10.1007/s00204-023-03578-1

**Published:** 2023-08-18

**Authors:** Jens Rengelshausen, Isabella Randerath, Thomas Schettgen, Andre Esser, Andrea Kaifie, Jessica Lang, Thomas Kraus, Patrick Ziegler

**Affiliations:** Institute for Occupational, Social and Environmental Medicine, Medical Faculty, RWTH Aachen University, Pauwelsstraße 30, 52074 Aachen, Germany

**Keywords:** Polychlorinated biphenyls, Occupational exposure, HELPcB

## Abstract

After the detection of high environmental and occupational exposure to polychlorinated biphenyls (PCBs) in a German recycling company for transformers and capacitors in 2010, the multidisciplinary medical surveillance program “HELPcB” (Health Effects in High-Level Exposure to PCB) was established for former PCB-exposed workers of the company, their family members, employees of surrounding companies, and area residents to investigate potential adverse health effects by PCB exposure in a longitudinal study approach with up to seven examination time points between 2010 and 2019. More than 300 individuals were enrolled into the program. Assessments particularly included plasma and urine concentrations of PCB congeners and their metabolites, clinical laboratory parameters, Comet assay, analysis of telomere length, neuropsychological examinations, psychological screening, abdominal and thyroid ultrasound examination. This review summarizes the main results of the studies conducted in the HELPcB program yielding relevant new data on potential adverse effects of PCB exposure in humans and potential mechanisms that underlie these effects. Even larger studies in PCB-exposed individuals are warranted to confirm the results of this program and to further establish causality between PCB exposure and clinical effects in humans.

## Introduction: polychlorinated biphenyls

Polychlorinated Biphenyls (PCBs) are organohalogen compounds that contain a biphenyl framework to which 1–10 chlorine atoms are attached, differing in position and number of chlorine atoms (World Health Organization 1976). There are a total of 209 different PCB congeners. A unique number has been assigned to each PCB congener as recommended by the International Union of Pure and Applied Chemists (IUPAC) which ranges from 1 to 209. Therefore, it has become common practice to use IUPAC numbers to identify individual PCB congeners. Chemical properties and toxicity of PCBs are determined by the number and position of chlorine atoms (1–10) that substitute hydrogen atoms in phenyl rings (Ballschmiter and Zell 1980). Depending on the number of chlorine atoms, PCBs can either be classified as higher chlorinated PCB congeners (HPCB), which contain six or more than six chlorine atoms, or as lower chlorinated PCB congeners (LPCB), which contains less than six chlorine atoms. There are 12 dioxin-like PCB congeners (dlPCB) with none or only one chlorine atom in ortho-position to the biphenyl ring that act on the aryl hydrocarbon receptor (AHR), similar to dioxin (also known as 2,3,7,8-tetrachlorodibenzo-p-dioxin) (Safe 1994).

About 70 PCB congeners are found in commercial mixtures. It has been a common practice to analyze only six congeners (so called “indicator congeners”) in environmental media that were regarded to be representative for the different mixtures, namely PCB 28, PCB 52, PCB 101, PCB 138, PCB 153, and PCB 180. Many chemical properties of PCBs made them useful in a variety of applications, including flame retardancy, extremely low electrical conductivity, strong plasticizing characteristics, such as in ceiling composites, and high aging resistance (Fishbein 1974; Kimbrough 1987). During the 1960s, Monsanto marketed PCBs under the name “Aroclor”, while Bayer marketed them under the name “Clophen”. Their applications ranged from open systems (paints, plasticizers, sealants, hydraulic oils) to closed systems (dielectrics in capacitors and transformers). In 1992, Ivanov and Sandell estimated that over one million tons of PCBs were produced worldwide (Ivanov and Sandell 1992). Production and trade of PCBs have been banned in Germany since December 1989 and worldwide since 2000 (Choi et al. 2008; Kalantzi et al. 2001; Ueno et al. 2003).

The International Agency for the Research for cancer (IARC) initially classified PCBs as “probably carcinogenic to humans” (Group 2a) but upgraded them to Group 1 (“carcinogenic to humans” in 2013) (Lauby-Secretan et al. 2013). Carcinogenic effects are attributed largely to the metabolism of parent PCBs (Lauby-Secretan et al. 2013). It is important to note that the German Research Foundation (DFG) distinguishes between higher and lower chlorinated PCBs in their assessment (DFG 2012). In contrast to the IARC, for the DFG, PCBs are (non-genotoxic) Category 4 human carcinogens, indicating that DNA damage is not the primary mechanism of action for carcinogenesis, suggesting a threshold for their effects. Thus, the DFG evaluated an occupational limit value (MAK, 3000 µg/m^3^ for the sum of indicator PCBs) as well as a biological limit value (BAT, 15 µg/L plasma for the sum of indicator PCBs), at which no contribution to cancer risk of workers can be anticipated.

PCBs are metabolized sequentially into monohydroxy and dihydroxy metabolites (OH-PCBs), with lower chlorinated PCBs being metabolized more rapidly than higher chlorinated PCBs (Hovander et al. 2002). According to Grimm and colleagues, PCB congeners can theoretically produce 837 monohydroxylated products (Grimm et al. 2015). Higher chlorinated biphenyls are chemically inert, and therefore do not degrade when exposed to acids, alkalies, or oxidizing agents. As a result of their hydrophobicity, they accumulate in the fatty tissues of organisms. Higher chlorinated PCBs can, therefore, be detected in the general population as a result of general nutrition (Schettgen et al. 2012b). As a result of biotransformation and worldwide spread, PCB mixtures used in transformer oil, capacitors, paints, or joint compounds can be very different from the PCB congeners found as background contaminants in blood and food. Contamination with non-background PCB congeners is relatively rare and is primarily associated with the disposal of toxic waste. In addition to inhaling or uptake via food, there is a particular risk of skin absorption in this situation (DFG 2012).

## Envio recycling Germany

PCBs have been used in electrical appliances, but since their use has been banned, PCBs must be thoroughly disposed to prevent further pollution. As a waste management company in Germany, Envio recycling had an approved process for dismantling PCB-contaminated transformers and capacitors with a global catchment area for its waste collection services (Hiester and Rademacher 2011). As a result of high commodity prices, PCB containing transformers have been shipped to Envio in Dortmund from the underground landfill Herfa-Neurode, where transformers had been finally stored (Hiester and Rademacher 2011).

A common local network transformer (20 kV/400 V) contained approximately 300 L of a mixture of oil and PCB. There were several thousand liters of the mixture stored in transformer stations at power plants (Bonk et al. 2011). It is estimated that Envio recycling disposed 4,500 tons of PCB-containing equipment every year in Germany (Hoffmeister et al. 2011).To clean PCB-contaminated transformer parts, Envio used a low-temperature rinsing process with tetrachloroethylene (“LTR2 process: Low Temperature Rinsing and Re-Use/Recovery”) (Hiester and Rademacher 2011). The cleaning of transformer liquids started with the removal of PCB-containing oils, followed by the washing and drying of transformer housings using tetrachloroethylene. Separation of PCBs from cleaning agents was achieved through downstream distillation. Besides processing the PCB distillation fraction, PCB-containing oils were also disposed of by disassembling metal sheets from the transformer core, with copper and aluminum coils being collected as secondary raw materials (Hiester and Rademacher 2011). A single hall has been approved by the supervisory authorities for the treatment of PCB-contaminated transformers at Envio. Transformers that were low polluted or clean were processed in a separate building. Additionally, low-polluted materials could be stored on the premises of the company in a tent. In spite of this, PCB-contaminated transformer parts and capacitor sheets were stored in an area by Envio, where only low-polluting capacitors were allowed to be processed (Hiester and Rademacher 2011).

Unauthorized storage of highly contaminated transformers and the improper handling of PCB containing binders then caused an increase in PCBs to be released in the Dortmund harbor area (Hoffmeister et al. 2011), which has also exposed many employees to PCBs. An environmental monitoring program conducted in the bay area between 2006 and 2008 repeatedly detected elevated levels of PCBs. In the end, it was determined that Envio recycling was the source of the PCB contamination found during the investigation (Bruckmann et al. 2011; LANUV 2012; Radermacher et al. 2011). A human biomonitoring study was, therefore, conducted in spring 2010 among staff members as well as their immediate family members to determine the magnitude of the risk of exposure (Kraus et al. 2012). In a gas chromatography/mass spectrometry (GC/MS) analysis, up to 236 µg /L plasma for the sum of the three lower chlorinated standard PCBs PCB 28, PCB 52, and PCB 101, as well as three of the higher chlorinated PCBs (PCB 138, 153, and 180), as well as six dioxin-like PCBs were identified, with the highest concentrations measured to date in Germany (Schettgen et al. 2012b). As a result of inadequate occupational health and hygiene precautions, the facility was forced to close for the handling of hazardous waste such as capacitors and transformers contaminated with PCBs (Kraus et al. 2012).

As a further consequence, the multidisciplinary medical surveillance program “HELPcB” (Health Effects in High-Level Exposure to PCB) was established for former PCB-exposed workers of Envio, their family members, employees of surrounding companies, and area residents to investigate potential adverse health effects by PCB exposure in a longitudinal study (Kraus et al. 2012). Individuals were admitted to the program if one of the following criteria was present:

- Serum concentrations of either one of the lower chlorinated PCBs were > 0.1 µg/L
- One of the higher chlorinated PCBs or the sum of the six indicator PCBs was above the reference value as derived by the German Human Biomonitoring Commission (DFG 2012)
- One of the dioxin-like PCBs was above the 95th percentile as reported by Schettgen et al 2011

**Fig. 1:**
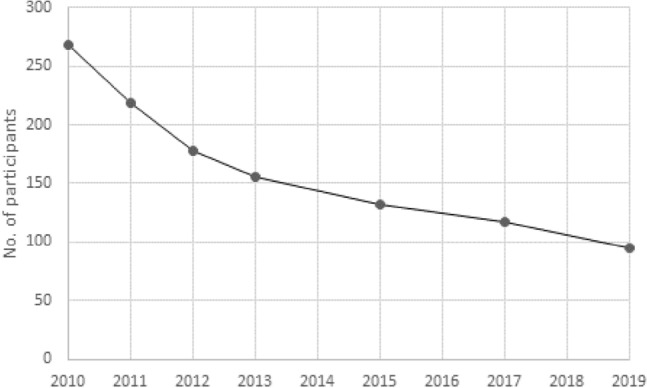
Number of participants at the seven examination time points of the HELPcB program

Systematic examinations of the participants started in September 2010 and included up to seven examination time points per individual until March 2020 with the following assessments: PCB exposure, medical and occupational history, physical examination, clinical laboratory parameters, Comet assay, antioxidative status, analysis of telomere length, neuropsychological examinations and neurography, psychological screening, abdominal and thyroid ultrasound examination, and pulmonary function tests (Kraus et al. 2012). A total of 363 individuals were enrolled into the program between 2010 and 2013. The number of participants at each of the seven examinations time points is shown in Fig. 1.

## PCBs and their metabolites in the HELPcB cohort

While PCBs are generally considered to be very stable, and they can only be metabolized in humans up to a limited extent, their metabolites have become of increasing interest for toxicology in recent years. In general, PCBs must be metabolized by cytochrome p450 enzymes through hydroxylation and epoxidation, with lower chlorinated PCBs being metabolized more rapidly than those with a higher chlorination level. According to the proposed mechanism, the hydroxyl group can be added directly to the biphenyl ring, or indirectly via an intermediate arene oxide through a 1, 2-shift (Hovander et al. 2002). Moreover, it is important to note that intermediately formed arene oxides have strong electrophilic properties and can bind to proteins, RNA, and DNA. In addition, there is a certain low level of OH-PCBs in blood as a result of their enterohepatic circulation. Therefore, determining the metabolite dose in the blood is far closer to the potential toxicological effect of PCBs than quantifying individual congeners in PCB-exposed individuals.

Aside from regular PCB testing in the HELPcB cohort, we, therefore, also analyzed and quantified hydroxy-PCBs (Quinete et al. 2015). There were only a few reports exploring state-of-the-art liquid chromatography with tandem mass spectrometry (LC–MS/MS) techniques for detecting OH-PCBs at the time the study was initiated. In most cases, sample preparation involved liquid–liquid extractions, back extractions, and evaporations. This led us to develop a novel, sensitive, and high-throughput solid-phase extraction method (SPE) coupled with tandem mass spectrometry (MS/MS). As a result of our approach to sample preparation and analysis, we were able to detect several different OH-PCB congeners in the same plasma sample subsequently reducing the amount of sample needed from each individual (Quinete et al. 2015). In addition, this method proved to be substantially more sensitive than previous LC–MS methods (Letcher et al. 2005; Dhakal et al. 2012; Tobiishi et al. 2011) and comparable to recent LC–MS/MS methods, gas chromatography with an electron-capture detector (GC-ECD) methods and gas chromatography with tandem mass spectrometry (GC–MS/MS) methods. This method, along with a number of internal standards, allowed us to detect different hydroxy-PCBs in plasma samples of highly exposed individuals, including low-chlorinated 4-OH-CB3, 4-OH-CB9, 4-OH-CB15, 4-OH-CB18 and 3′-OH-CB28 5-OH-CB28 + 4′-OH-CB25 + 4-OH-CB31, tetra-chlorinated 4-OH-CB61 and 4-OH-CB76 as well as pentachlorinated 4-OHCB101, 3- OHCB101, 4-OHCB101, 3-OHCB101 and 4-OH-CB107, 4-OH-CB108, 3-OH-CB118, 4-OH-CB130, 3-OH-CB138, 4-OH-CB146, 3-OH-CB153, 4-OH-CB172, 3OH-CB180, and 4-OH-CB187 (Quinete et al. 2015). It is important to note that four metabolites of PCB 28 (along with two isotopically labeled internal standards) were predicted, custom synthesized, and identified for the first time in humans (Quinete et al. 2017). In comparison to the respective parental PCB concentrations of 0.33–272 ng/mL, the concentrations of the OH-PCBs ranged from 0.11 to 75.4 ng mL1. Further, several peaks of unknown chlorinated OH-PCB congeners were detected, showing the necessity for the development of new analytical standards.

As mono- and di-chlorinated congeners are rapidly metabolized and might evade classical biomonitoring of PCBs or PCB metabolites in plasma, the (SPE) to LC–MS/MS method was further developed for the quantification of hydroxylated PCBs in urine as a biomarker of PCB exposure (Quinete et al. 2016). Urine is a useful source for biomarker investigation, since it is readily available and collected by less invasive methods than blood (plasma and serum). Urine samples enabled the detection of trace amounts of hydroxylated PCBs, as low as 0.01 ngmL-1, and reduced matrix effects in comparison to plasma. We found that in urine samples from the HELPcB cohort, the predominant congener was 3′-OH-CB28 (Quinete et al. 2016). Urinary PCB metabolite levels were also found to exhibit a strong correlation with body burden of PCBs and metabolites in plasma, and were higher in PCB-exposed groups than in non-exposed individuals. Additionally, levels of OH-PCBs in urine declined over time following the same pattern as in plasma (Quinete et al. 2016).

## Elimination kinetics of PCB congeners from the human body

The HELPcB surveillance program included blood sampling from the participants at each of the visits (Kraus et al. 2012). Blood samples from up to six cross-sectional examinations between 2010 and 2017 were analyzed for the six non-dioxin-like indicator congeners (PCB 28, PCB 52, PCB 101, PCB 138, PCB 153, PCB 180), eight dioxin-like congeners (PCB 105, PCB 114, PCB 118, PCB 123, PCB 156, PCB 157, PCB 167, PCB 189), and three additional non-dioxin-like congeners (PCB 66, PCB 74, PCB 99)to determine the elimination kinetics after occupational exposure (Esser et al. 2021).

Higher chlorinated PCB congeners (e.g., PCB 138, PCB 153, PCB 180) have been shown to persist in the environment and accumulate in the food chain leading to a continuous intake and a background burden which can be detected in the plasma of the general population and which is increasing with age (Schettgen et al. 2015). In contrast, lower chlorinated PCB congeners (e.g., PCB 28, PCB 52, PCB 101) can hardly be detected in the plasma of individuals who do not have a specific occupational or indoor exposure to PCBs (Schettgen et al. 2012a). These characteristics of higher and lower chlorinated PCBs need to be considered when investigating the elimination of PCBs from the human body. PCBs are highly lipophilic compounds, and therefore accumulate in adipose tissue (Bourez et al. 2013). The total body burden of PCBs was shown to be higher in obese than in lean individuals, and drastic weight loss led to increased serum concentrations of PCBs (Kim et al. 2011). Therefore, PCB plasma concentrations have to be either adjusted for total blood lipids or transformed to total body burden of PCBs before estimating the elimination kinetics (Esser et al. 2021).

Two major categories of biomonitoring data have been used to characterize the elimination kinetics of PCB congeners in humans: longitudinal data with sequential blood samples from the same individual and cross-sectional data with only one sampling point in many individuals (Ritter et al. 2011). Longitudinal data are preferred for estimating the elimination half-lives of PCBs because individual changes over time can be taken into consideration. However, the observed decline phase of persistent compounds like the higher chlorinated PCBs is confounded by the continuous intake from ongoing exposure and changes in body composition over time (Milbrath et al. 2009). In this case, an estimated elimination half-life directly based on observed plasma concentrations over time is called “apparent half-life” in contrast to the “intrinsic half-life” which just refers to the elimination processes in the human body. The intrinsic half-life of a compound may be approximated using methods that account for ongoing exposure and changes in body composition (Ritter et al. 2011).

The influence of ongoing background exposure can be addressed by investigating individuals that have had a high occupational or accidental exposure to PCBs for a defined time period (Shirai and Kissel 1996). Background exposure becomes negligible especially during the initial decline phase of the exposure. In addition, the positive correlation of background plasma concentrations of higher chlorinated PCBs with age (Schettgen et al. 2015) can be used for estimating an age-dependent share of PCB plasma exposure and for deriving age-adjusted PCB plasma concentrations. Elimination half-lives estimated from these concentrations are regarded to reflect the intrinsic half-life of the respective PCB congener (Esser et al. 2021).

**Fig. 2:**
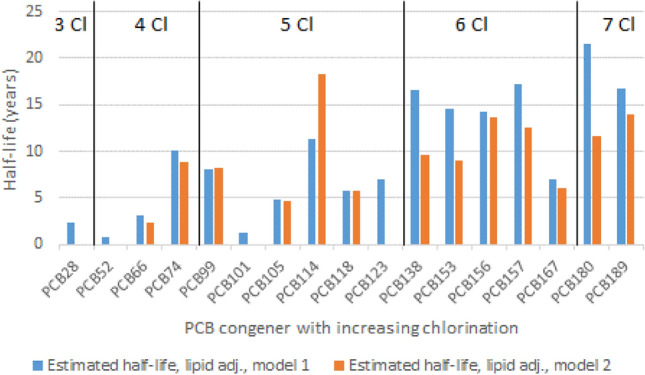
Estimated apparent and intrinsic half-lives of PCB congeners with increasing chlorination

The longitudinal design of the HELPcB program with blood samplings from up to six sequential examination visits per participant has enabled an extensive assessment of the elimination kinetics of 17 PCB congeners. Confounding by background exposure was limited because only individuals with PCB exposures that exceeded pre-defined lower limits were included into the cohort reflecting an occupational or accidental exposure to PCBs (Kraus et al. 2012). Nevertheless, elimination half-lives of the PCB congeners were estimated based on two different models to further differentiate between apparent and intrinsic half-lives. Model 1 was based on the decay model of Friedman with the addition of a nested linear regression model to account for multiple sequential data points. Apparent half-lives were calculated separately for PCB plasma concentrations, lipid adjusted plasma concentrations, and total body burden of PCB. Model 2 included age-adjusted PCB plasma concentrations instead and hence yielded an approximation of intrinsic half-lives of the PCB congeners (Esser et al. 2021). The results are summarized in Fig. 2.

The estimated apparent half-lives of the PCB congeners (model 1) varied over a wide range from 1 to 18 years. The estimated intrinsic half-lives (model 2) appeared to be shorter for most of the higher chlorinated PCB congeners supporting the concept of long environmental persistence and ongoing intake. Higher chlorinated PCB congeners also showed a trend for longer intrinsic half-lives compared to the lower chlorinated PCB congeners pointing to a slower metabolism in and/or excretion from the human body. The half-life estimates based on total blood lipids adjusted or total body burden transformed PCB concentrations did not differ in a relevant manner from the values based on pure plasma concentrations of the PCB congeners in both models. In addition, the elimination half-lives of the main hydroxy metabolites of the PCB congener PCB 28 have been investigated on a subset of data including four plasma sampling points over a time period of 4 years (Quinete et al. 2017). While the elimination half-life of PCB 28 was estimated at 4.32 years (95% CI 2.95–8.12 years), the elimination half-lives of four hydroxyl metabolites varied between 2.77 years (95% CI 2.20–3.65 years) for 3′-OH-PCB 28 and 7.55 years (95% CI 6.37–9.27 years) 3-OH-CB 28 indicating a potential enterohepatic circulation of low-chlorinated PCB metabolites (Quinete et al. 2017).

Previous studies on elimination half-lives have included either adult individuals with high occupational PCB exposure and one longitudinal follow-up after 5 years (Aylward et al. 2014) or even after 28 years (Seegal et al. 2011) or adult individuals with general environmental exposure in a cross-sectional design (Ritter et al. 2011). Two further longitudinal studies have investigated PCB concentrations in children (Grandjean et al. 2008; Wimmerová et al. 2011). The results on the elimination half-lives of PCB congeners from these previous studies are generally in line with the findings from the most current study (Esser et al. 2021). However, the half-life data presented in Fig. 1 are based on a longitudinal study with more sequential data points and more PCB congeners than the respective data from previous studies. Numerical differences between the elimination half-lives of PCB congeners estimated from the studies are quite substantial (about twofold to threefold) and may be related to differences in exposure levels of the study populations, time points of blood sampling, bioanalytical methods, and kinetic models for the final estimation of the elimination half-lives. Taking all studies together, PCB congeners showed a long persistence in the human body with elimination half-lives in the range of 1–20 years which tended to be about twofold longer in the higher chlorinated PCB congeners (≥ 6 chlorine atoms) compared to the lower chlorinated PCB congeners (≤ 5 chlorine atoms).

## Effects of PCBs on telomeres and expression of telomerase

Telomeres are highly repetitive nucleotide sequences at the end of chromosomes. They protect chromosomes from erosion and fusion and are important for maintaining genomic stability (Blasco 2005). In normal somatic tissues, telomere length decreases with aging in vitro and in vivo, and therefore reflects the proliferative history of somatic cells (de Lange 1998). Critically shortened telomeres have been associated with replicative exhaustion and tissue failure (de Lange 1998). Several tissues, including cells of the germline as well as embryonic and adult stem cells are able to protect themselves against telomere shortening by expressing telomerase (*htert*), the telomere extending enzyme (Flores et al. 2006). Furthermore, activated T cells respond to antigenic stimuli with clonal proliferation, thereby equally limiting telomere attrition by upregulating *htert* expression (Hiyama et al. 1995). The ability of T cells to reactivate telomerase declines after each round of stimulation and telomerase expression levels are increasingly insufficient to maintain telomere length (TL). Telomerase expression levels are, therefore, believed to control the lifespan of T cells (Roth et al. 2003).

When we measured the TL of individuals of the HELPcB cohort in 2011, we found that exposure to PCBs causes premature telomere loss in peripheral blood lymphocytes (Ziegler et al. 2012), but not in granulocytes. Indeed, granulocytes displayed a normal age-adjusted TL distribution in PCB-exposed individuals. Granulocytes are post-mitotic cells, whose TL reflects the proliferation history of the hematopoietic stem and progenitor cells (HSPCs) they are derived from (reviewed in (Vasko et al. 2018). These findings have been shown by us and other researchers in normal individuals, including frequent blood donors (Scheding et al. 2003; Rufer et al. 1999) and in certain model diseases with enhanced hematopoietic stem cell renewal (Ziegler et al. 2012). Therefore, the observation that no telomere attrition was observed in granulocytes argued against increased telomere consumption in the underlying HSPC compartment in the PCB-exposed individuals within the HELPcB cohort (Ziegler et al. 2017).

In contrast, several studies reported that organochlorine pesticides and PCBs were positively and significantly associated with TL in leukocytes (LTL). TL was increasing across low doses of exposure to persistent organic pollutants (POPs) with affinity to the aryl hydrocarbon receptor (AHR) (Shin et al. 2010). Because many dioxin-associated cancers are also associated with longer LTL, these results may provide insight into the mechanisms underlying PCB- and dioxin-related carcinogenesis (Shin et al. 2010). Further studies which assessed the association between PCBs, dioxins and furans and leukocyte TL (LTL) among 1330 or 2431 US adults (Mitro et al. 2016; Scinicariello and Buser 2015) showed that exposures to non-ortho PCBs and toxic equivalency were associated with 3.74% and 5.29% longer LTLs, respectively (Mitro et al. 2016). The highest quartile of exposure was associated with 9.16% and 7.84% longer LTLs (Mitro et al. 2016). Non-dioxin-like PCBs were not associated with LTL.

This distinction from our study may be attributed to factors such as specific properties of the subjects in the HELPcB study, e.g., the abundance of lower chlorinated PCBs, the extent of PCB contamination (concentrations on average 5 × higher than in the plasma of the background population), and methodical differences concerning telomere measurement (differentiation between granulocytes and lymphocyte TL using flow cytometry versus determination of total LTL by polymerase chain reaction).

As we found that PCBs causes premature telomere loss in peripheral blood lymphocytes, we later recognized that the rate of telomere shortening of PCB-exposed individuals depends both on the PCB-congener profile as well as on the concentration of PCBs found in the individuals` plasma (Ziegler et al. 2017). A high plasma concentration of lower chlorinated PCBs led to more accelerated telomere shortening than a low concentration. Most importantly we could not detect a significant correlation between TREC levels and the sum of PCBs, suggesting the loss of telomeric DNA in T cells during their activation and antigen stimulated proliferation. In addition, when used in a lymphoproliferation assay, blood plasma of PCB-exposed individuals inhibited the expression of telomerase, the telomere extending enzyme. Finally, 3-OH-CB28, a metabolite of PCB 28, detected in the blood plasma of PCB-exposed individuals, inhibited telomerase expression when used as a single agent and accelerated telomere shortening in long-term in vitro cell expansions studies (Ziegler et al. 2017). We later on confirmed the effects of PCB 28 metabolites on telomerase gene expression using reporter-gene assays and determined a lowest observed adverse effect level (LOAEL) at a plasma concentration between 0.5 and 2 µg/L of lower chlorinated PCBs (Vasko et al. 2018).

These findings are in line with a longitudinal study from Finland including double measurement of TL at two time points in a cohort of more than 1000 subjects. They found that oxychlordane, trans-nonachlor, and PCB 153 were significant predictors of telomere attrition in participants (Guzzardi et al. 2016). In addition, Yuan et al. (2018) report that high levels of aging-related diseases were associated with POP exposure, including 25 different PCB congeners. Molecular-level experimentation in this study found that telomere dysfunction, hypomethylated DNA modification, and systemic chronic inflammation may have led to the development of these diseases. Finally, in vitro studies with liver and keratinocyte cell lines exposed to low-chlorinated PCB congeners showed a shortening of TL based on inhibition of *hTERT* expression as well as reduced enzyme activity (Jacobus et al. 2008; Senthilkumar et al. 2011, 2012).

A follow-up assessment of TL within individuals of the HELPcB cohort in 2017 demonstrated that although the age-adjusted TL of lymphocytes remained shortened as compared to 2011, it appeared to be recovering (Beier et al. 2021)**—**with the exception of CMV seropositive more highly exposed individuals. In this group of participants, lymphocyte TL remained significantly shortened when the levels of lower chlorinated PCBs measured in 2011 were above the median. In addition, a mixed effects model analysis showed significant influence of the respective PCB levels, smoking, and the interaction of PCB levels and CMV status. A recovery of TL in the group of CMV positive participants with a respective PCB load above the median was barely or even not observed (Beier et al. 2021).

**Fig. 3:**
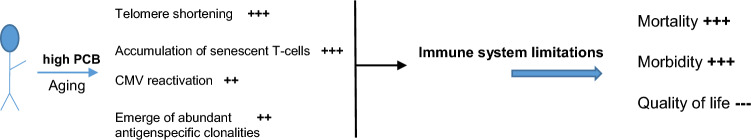
Proposed mechanism of high PCB exposure on telomere length and potential clinical effects

Taken together our results from the measurement of TL in the HELPcB cohort, we propose a mechanism in which PCB sustain telomere attrition during aging in CMV positive individuals and enhance functional limitations of the immune system: telomere shortening provides the counting mechanism for replicative senescence, and persistent infections such as cytomegalovirus (CMV) or Epstein–Barr virus (EBV) repeatedly induce the expansion of the responding memory CD8 + T cell compartment for the lifetime of an individual. The continuous exposure to antigen and the subsequent replicative stress makes virus-specific CD8 + T cells susceptible to age-dependent functional exhaustion. In PCB-exposed individuals, where T cells go through rounds of expansion presumably without restoration of TL, accelerated telomere shortening of virus-specific memory T cells could, therefore, cause higher rates of immune exhaustion after shorter time of infection. We, therefore, hypothesize that PCBs through the inhibition of telomerase gene expression accelerate replicative senescence and promote immunological dysfunctions and loss of virus-specific memory CD8 + T cells (Fig. 3).

## Genotoxic effects of PCB 28 metabolites

Previous research on genotoxicity of PCB congeners that we also measured in our HELPcB cohort resulted in contradictory findings. JECFA (2016) noted that 14 in vitro genotoxicity experiments were conducted on the PCB congeners PCB 52, 101, 138 and 153 and 3 in vivo genotoxicity experiments were conducted on the PCB congeners PCB 52 and 153. It was reported that 10 of the 14 in vitro genotoxicity experiments gave a positive result (2 experiments on PCB 52, 2 experiments on PCB 101, 1 experiment on PCB 138, and 5 experiments on PCB 153), while 4 of the experiments (3 experiments on PCB 52 and 1 experiment on PCB 138) gave negative results. (JEFCA 2016) A total of three in vivo experiments were conducted, two with PCB 52 and one with PCB 153, and no genotoxic findings were reported in any of them. Based on similar investigations examined by IARC (Lauby-Secretan et al. 2016), “the absence of genotoxic activity for PCB 153 cannot be established with certainty”, and based on the results, “the Working Group concluded that the results do not establish a definitive conclusion” with respect to PCBs 15, 47, 52, 77, 101, 118, 138, 155, and 180. As we detected PCB 28 and its hydroxy metabolites at high levels in the blood plasma of individuals of the HELPcB cohort, we performed genotoxicity testing of 3′-OH-PCB 28 and 5-OH-PCB 28, the major PCB 28 metabolites, by means of Comet assay and H2Ax phosphorylation (Vasko et al. 2018). Both metabolites were found to be genotoxic in immortalized cell lines as well as in proliferating primary T-lymphocytes. Genotoxicity could be found for the metabolites at a concentration of 10 µM, with parental PCB 28 showing no such effect. The extend of DNA fragmentation correlated with the duration of the incubation period and increased dependent on concentration, especially for 3′-OH-4′,4,6′-trichlorobiphenyl. The DNA repair response induced by PCB metabolites showed a linear dose–response relationship in all cells tested.

The DNA damage shown for PCB 28 metabolites could be caused primarily by adduct formation with PCB derivatives or by secondary adducts such as oxidized fatty acids (Schilderman et al. 1999; Zhao et al. 2004). Using the “32P post-labelling” method, DNA adducts were demonstrated in the hepatopancreas of crayfish from PCB-polluted waters. The number of PCB 28 adducts and other LC PCB adducts could be linked with PCB concentration (Schilderman et al. 1999). For this purpose, isolated DNA and PCBs metabolized by rat microsomes were incubated in the presence of H2O2 and myeloperoxidase. Subsequently, four different adducts were detected for PCB 28 by 32P post-labeling (Schilderman et al. 2000).

It is important to note that the enzymes involved in the catalysis of PCBs, such as the CYP450 enzymes, peroxidases, and prostaglandin H synthase, are widespread in human tissues and organs, but mainly found in the liver. Due to the distribution of low-chlorinated (hydroxy-) PCBs throughout the body, further oxidation (biochemically or non-enzymatically catalyzed) depends on the specific, enzymatic equipment of the cells reached by PCB 28 and its hydroxy metabolites and can vary considerably.

## Effects of PCBs on depressive symptoms and neuropsychological functions

Occupational or environmental exposure to PCBs and the resulting PCB body burden have been associated with the disturbance of neuropsychological functions in humans. A positive age-dependent association between PCB body burden and fine motor performance has been observed (Haase et al. 2009). Similarly, PCB body burden has been associated with a decrease in visual motor coordination and hand steadiness (Schantz et al. 1999). In a case–control study, occupational exposure to PCBs was related to neurobehavioral dysfunction which was improved following a detoxification program. Self-appraisal scores for depression, anger, and fatigue were elevated in the exposed workers and remained unchanged after detoxification (Kilburn et al. 1989). In elderly people (55–74 years), who were environmentally exposed to PCBs, an increase in total serum PCB concentrations was associated with a significant increase in depressive symptoms measured by the Beck depression inventory (Fitzgerald et al. 2008).

The HELPcB cohort of individuals occupationally or accidentally exposed to PCBs has offered the opportunity to further investigate the potential relationship between PCB body burden and disturbances of (neuro-) psychological functions (Kraus et al. 2012). The PCB body burden was determined by the plasma concentrations of the six indicator PCBs and eight dioxin-like PCB congeners. Three sum variables were derived thereof: lower chlorinated PCBs (LPCB = PCB 28, PCB 52, PCB 101), higher chlorinated PCBs (HPCB = PCB 138, PCB 153, PCB 180), and dioxin-like PCBs (dlPCB = PCB 105, PCB 114, PCB 118, PCB 123, PCB 156, PCB 157, PCB 167, PCB 189). The sum variables were then split at the 95th percentile of a reference population with no additional occupational exposure to PCBs (Schettgen et al. 2011). Study participants were assigned to have a high PCB body burden with sum variables above the 95th percentile compared to participants with sum variables below this threshold. The latter were assigned to the group of normal PCB body burden (Gaum et al. 2014).

As a first approach, the health-related quality of life (HRQL) and the quality-adjusted life years (QALY) were assessed in relation to PCB exposure at three consecutive examination time points separated each by 1 year using the EQ-5D-3L questionnaire to determine HRQL (Rabin and de Charro 2001). QALY was calculated as the product of HRQL and the remaining average life expectancy (Esser et al. 2014). Subjects with high HPCB body burden showed a significant decline in QALY over time compared to subject with normal HPCB burden who had stabilized QALY after the second study year (Esser et al. 2015).

As a second step, the severity of mental symptoms was assessed by four subscales of the German Version of the Patient Health Questionnaire (PHQ-D) and set into relation to the PCB body burden. The respective subscales measured participants’ mental state regarding somatoform syndrome, depressive syndrome, anxiety syndrome, and panic syndrome. Linear relationship between PCB body burden and these syndromes was assessed by multiple regression, controlling for age, gender, education, and job status. A significant correlation between all three PCB sum variables and the category depressive syndrome was observed. Prevalence rates for the category depressive syndrome were higher in participants with high PCB body burden compared to participants with normal body burden for all three PCB sum variables. General conclusions on these results are limited by the relatively small sample size (*n* = 136) (Gaum et al. 2014).

An effect of PCB exposure on the prevalence of depressive symptoms may be directly triggered by the knowledge of a potential health hazard leading to an increased psychological strain (Shaw et al. 1999). Alternatively, an indirect effect mediated by an impact on neurotransmitters may be postulated. Low dopamine concentrations in the CNS have been associated with different types of depression and depressive symptoms (Dunlop and Nemeroff 2007). Acute exposure to PCBs in non-human primates resulted in reduced dopamine concentrations in the caudate, putamen, and hypothalamus (Seegal et al. 2010). Furthermore, occupational exposure to PCBs was related to reduced dopamine transporter density in the basal ganglia of the CNS in women but not in men as assessed by radiolabeled neuroimaging (Seegal et al. 2010).

In the HELPcB cohort, potential changes in the central dopaminergic system in human after PCB exposure were assessed by measuring the concentrations of homovanillic acid (HVA) as the major dopamine metabolite in urine with normalization on urine creatinine (crea) concentrations (Putschögl et al. 2015). Similarly, potential alterations in the central neurotransmitter norepinephrine were investigated by the urine concentrations of the norepinephrine metabolite vanillylmandelic acid (VMA). Longitudinal samples from three time points, each separated by 1 year, were included into the analysis. In line with previous study results, a cross-sectional analysis showed a negative correlation between the plasma concentrations of all PCB congeners and the urine concentrations of HVA/crea at the first but not at the following sampling time points whereas negative correlations for only single PCB congeners and VMA/crea were observed at the second and third sampling time point. In a longitudinal analysis, only subjects with high body burden of HPCB had significantly lower urine concentrations of HVA/crea over time than subjects with normal PCB exposure (Putschögl et al. 2015). No significant effects were found for VMA/crea in this analysis.

Based on these results, the indirect impact of changes in the dopaminergic system in subjects with high PCB exposure on the occurrence of depressive symptoms was investigated. Plasma PCB and urine HVA/crea concentrations as well as depressive symptoms using the Beck’s depression inventory (BDI-II) were measured at the first two examination time points separated by 1 year (Gaum et al. 2017). The cross-sectional results for the tested mediations were non-significant. Longitudinally, the three sum variables of PCB congeners were negatively associated with urinary HVA/crea concentrations at the first time point and these reduced HVA concentrations were associated with increased depressive symptoms at the second time point 1 year later. Hence, the significant positive association between PCB body burden and depressive symptoms is partially mediated by reduced HVA/crea concentrations in urine which may reflect lower concentrations of dopamine in the CNS. For urinary VMA/crea, such effects were not found (Gaum et al. 2017).

In addition to effects on the neurotransmitter dopamine, studies on systemic stress hormone and thyroid hormone concentrations after PCB exposure yielded inconsistent results in previous studies. While an inverse association between PCB body burden and dehydroepiandrosterone sulfate (DHEAS) concentrations was observed in female workers during menopause (Persky et al. 2012), a positive association was found between PCB exposure and DHEAS concentrations in male employees (Persky et al. 2012; Sun et al. 2016). Similarly, a systematic review on potential effects of PCB exposure on thyroid hormone homeostasis has concluded that such an effect has not yet been convincingly shown but also cannot be excluded based on the available data (Hagmar 2003). Hence, potential cross-sectional and longitudinal effects of PCB exposure on stress hormones and thyroid hormones were assessed in the HELPcB cohort.

Regarding the impact of PCB concentration on stress hormones, plasma concentrations of PCBs using the three sum variables LPCB, HPCB, and dlPCB and plasma concentrations of (DHEAS) and cortisol were measured at three examination time points each separated by 1 year. Plasma concentrations of LCPB were positively correlated with DHEAS concentrations at each time point. Subjects with high LPCB body burden had a higher risk of elevated DHEAS concentrations than subject with normal LPCB body burden. A respective effect for cortisol was not observed (Gaum et al. 2020). For the assessment of thyroid hormones, plasma concentrations at four examination time points each separated by 1 year were included into the analysis. As a main result, HPCB body burden correlated negatively to the free T3 hormone concentrations cross-sectionally as well as longitudinally. In addition, a weak decrease in free T4 concentrations over time was observed in subjects with high exposure to HPCB or dlPCB. In these subjects, also an increase in TSH receptor antibody concentrations was identified over time. Other thyroidal antibody and morphological findings were inconsistent (Gaum et al. 2016).

Free thyroid hormone concentrations have been clinically associated with depression severity in previous studies (Berent et al. 2014; Hage and Azar 2012). Therefore, the interaction of PCBs with free T4 concentrations and its associations with HVA concentrations and depressive symptoms were investigated in the HELPcB cohort at three examination time points separated by 1 year using the PHQ-D questionnaire. Only LPCB concentrations impacted the association between free T4 and HVA consistently in the cross-sectional and the longitudinal analyses. Similarly, an interaction on the association between free T4 concentrations and depressive symptoms was only observed for LPCB concentrations. Hence, these results suggested that depressive symptoms after exposure to lower chlorinated PCBs may be related to dopamine and thyroid system alterations (Gaum et al. 2019). However, further mechanistic analyses are needed.

In addition to depressive symptoms, also neuropsychological functions were investigated in more detail in the HELPcB cohort. The following six categories of neuropsychological measures were assessed in a cross-sectional approach: intelligence, word fluency, flexibility, learning and memory, sensorimotor processing, and attention. In a multivariate statistical approach using structural equation modeling to account for interdependencies, a significant negative influence of total PCB body burden on word fluency and sensorimotor processing was found without a significant effect on attention and memory while controlling for age and education. More specifically, high LPBC burden had a negative effect on letter fluency with alternating and semantic fluency with single and alternating criterion and led to a stronger decrease of performance with longer duration of the fluency task (Fimm et al. 2017). These results might be related to decrease of cerebral dopamine by PCB exposure impacting verbal fluency and motor processing.

The hypothesis of a dopamine-related neurophysiological pathway mediating the influence of PCB exposure on fine motor performance was further investigated by measuring two dimensions of fine motor skills, accuracy (steadiness, line tracking) and speed (line tracking, aiming, tapping) in relation to plasma PCB concentrations and urine HVA concentrations in a cross-sectional manner. The body burden of LPCB, HPCB, and dlPCB was negatively correlated with steadiness accuracy and line tracking accuracy as well as with HVA/crea concentrations in urine. Multiple linear regression analyses revealed a significant direct path effect of LPCB, HPCB, and dlPCB body burden on line tracking accuracy. Indirect path effects of PCB body burden with moderation through HVA were found for line tracking accuracy and steadiness accuracy in line with the hypothesis that PCB body burden is associated to lower concentrations of HVA which is then associated with lower performance in the accuracy tests. Effects of PCB body burden on the speed-related tests were not observed (Gaum et al. 2021).

**Fig. 4:**
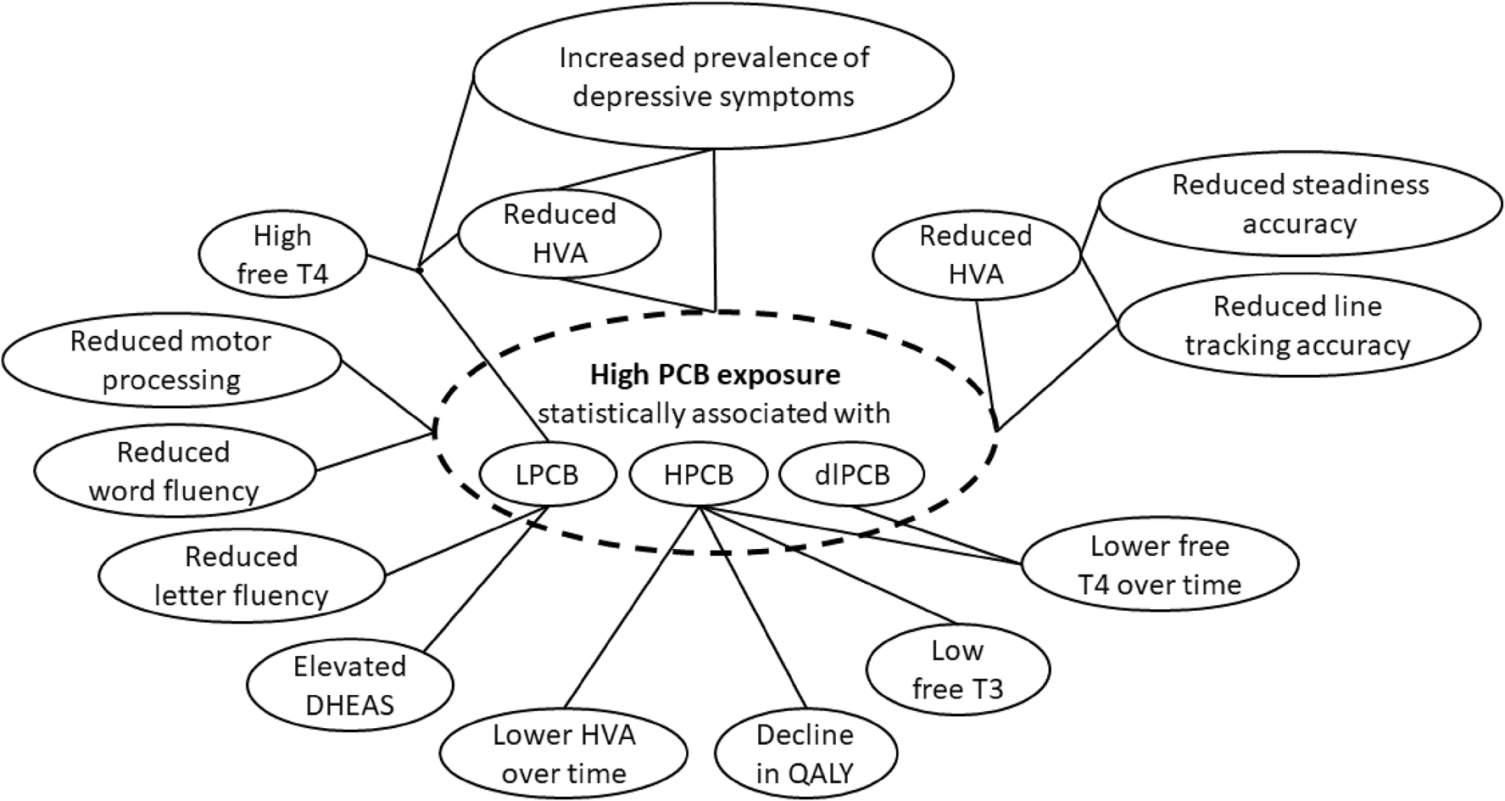
Statistical associations of PCB exposure with neuropsychological functions, depressive symptoms, neurotransmitters, and stress hormones. Confounding pathophysiologic factors were taken into account as far as possible. The associations do not mean causality until further proof

In conclusion, the investigations on the HELPcB cohort have yielded substantial insights into the effects of PCB exposure in humans on depressive symptoms and neuropsychological functions. They have also delivered a potential theoretical pathophysiological concept involving the dopamine neurotransmitter system as a mediator for the PCB-induced clinical effects. The respective associations are summarized in Fig. 4. Owing to the sample size (between 100 and 200 subjects from the HELPcB cohort) that these investigations are based on, further studies in PCB-exposed subjects would be needed to confirm the results summarized here and to further build the evidence for a potential causal relationship between PCB exposure and clinical effects in humans.

## Liver and skin abnormalities and diabetes mellitus: associations with occupational PCB exposure

Systemic exposure to exogenous chemicals like medicinal products may affect the liver in its tissue integrity and function and may lead to drug-induced liver injury (Hoofnagle and Björnsson 2019). PCB exposure has also been subject to extensive studies in animals in humans focusing on hepatobiliary endpoints like liver size, histopathology, and serum biomarkers of liver function and injury (Carlson et al. 2023). Metabolism of PCBs by enzyme systems in the liver has further supported the hypothesis that PCB exposure may lead to liver injury and impact liver function (Grimm et al. 2015). PCB exposure to rats led to increases in liver transaminase activities and histopathological changes in hepatic structure (Ounnas et al. 2016; Kutlu et al. 2007). The results from human studies were less clear so far. Increased PCB exposure in the NHANES cohort study was associated with activity elevations of serum alanine aminotransferase (ALT), aspartate aminotransferase (AST), and gamma-glutamyl transferase (GGT) (Cave et al. 2010; Serdar et al. 2014). However, the clinical consequences of these observations still need to be confirmed.

Potential adverse effects of PCB exposure on the liver were investigated at five examination time points between 2010 and 2015 in the participants of the HELPcB program by assessing serum biomarkers for liver injury and function and ultrasound imaging of liver size and structure (Kaifie et al. 2019). A longitudinal mixed model analysis controlling for alcohol consumption, medication, age, and total blood lipids revealed an inverse association for GGT activity as a dichotomized outcome variable and the sum of the non-dioxin-like indicator congeners and PCB156. The analysis further showed an association for liver size (normal/abnormal) and single PCB congeners. However, associations of PCB exposure with AST and ALT activities or with liver structure abnormalities could not be established. Also here, the clinical consequences of these findings still need to be elucidated and are subject to further surveillance of the participants in the HELPcB program.

Exposure to PCBs has been associated with the prevalence of diabetes mellitus in many cross-sectional and some longitudinal studies in environmentally exposed individuals and in occupationally exposed cohorts (e.g., Silverstone et al. 2012; Persky et al. 2012). Due to the large number of risk factors for diabetes, it is difficult to establish a causal relationship with PCB exposure. We have investigated the prevalence of diabetes mellitus, the proportion of glycol-hemoglobin A_1C_ (HbA_1C_), and the occurrence of insulin-related autoantibodies at four examination time points between 2010 and 2013 in the HELPcB cohort in relation to the individual PCB exposure (Esser et al. 2016). Using a logistic regression model, an elevated odds ratio for diabetes mellitus was found for the third quartile of PCB52 concentrations at the first examination time point. In addition, a multiple linear regression showed an influence of several PCB congeners on the HbA_1c_ values at the third and fourth examination time point. Due to the small cohort and the small number of diabetes cases in this cohort, the findings need to be interpreted with caution regarding causation.

A further target organ for adverse effects of PCB exposure is the skin (Carlson et al. 2023). Clinical evidence for this relation is mainly derived from two incidents of accidental PCB exposure by contaminated rice oil 1968 in Yusho, Japan, and 1978 in Yu-Cheng, Taiwan (Kanagawa et al. 2008; Masuda 1985; Guo et al. 1999). Chloracne and hyperpigmentation of the skin were among the most commonly observed skin reactions that were also correlated to plasma concentrations of PCB congeners in the affected patients (Mitoma et al. 2015). Both skin reactions are officially recognized as occupational disease following occupational PCB exposure in Germany (BMAS 2018).

In 2014, 92 participants of the HELPcB program underwent a dermatological screening examination to detect skin abnormalities in potential relation to PCB and dioxin exposure. In addition, dermatological screening for skin malignancies was performed in participants of the HELPcB program at seven examination time points between 2010 and 2016 (Leijs et al. 2018). Acneiform lesions and hyperpigmentation of the skin were the most common abnormalities observed in the skin of the participants. The occurrence of hyperpigmentation was correlated to the lipid adjusted plasma concentrations of PCB congeners as well as to the lipid adjusted serum dioxin concentrations of the participants confirming the relation of skin hyperpigmentation to PCB exposure. Skin cancer screening revealed five cases of confirmed and two cases of suspected basal cell carcinoma, two cases of malignant melanoma, and one case of cutaneous T cell lymphoma. Whether the incidence of these malignancies in the PCB-exposed cohort is higher than in the general population remains to be investigated in larger cohorts.

## Summary and conclusion

More than 10 years after the start of the multidisciplinary medical surveillance program HELPcB, the studies conducted in the program have yielded new scientific information on adverse effects of PCB exposure in humans and potential mechanisms that underlie these effects. The main findings are:

- A novel, sensitive, high-throughput solid-phase extraction method coupled with tandem mass spectrometry enabled detection of several different OH-PCB congeners as metabolites in plasma as well as in urine with higher sensitivity than previous LC–MS methods.
- Elimination half-lives of 17 PCB congeners ranged between 1 and 20 years and tended to be about twofold longer in the higher chlorinated PCB congeners (≥ 6 chlorine atoms) compared to the lower chlorinated PCB congeners (≤ 5 chlorine atoms).
- PCB exposure appeared to cause premature telomere loss in peripheral blood lymphocytes but not in granulocytes. The rate of telomere shortening in lymphocytes of PCB-exposed individuals depends on the PCB-congener profile and individual plasma PCB concentrations. This effect was not reversible in CMV seropositive individuals with higher PCB exposure.
- The two major hydroxy metabolites of PCB 28 were found to be genotoxic in immortalized cell lines and in proliferating primary T-lymphocytes at a concentration of 10 µM with PCB 28 itself showing no such effect.
- Participants with high HPCB body burden showed a significant decline in quality-adjusted life years (QALY) over time compared to subject with normal HPCB burden.
- Prevalence rates for the category depressive syndrome in the PHQ-D were higher in participants with high PCB body burden compared to participants with normal body burden.
- Only participants with high HPCB body burden had significantly lower urine concentrations of the major dopamine metabolite HVA than participants with normal PCB exposure. The association between PCB body burden and depressive symptoms appeared to be partially mediated by reduced HVA concentrations in urine potentially reflecting lower concentrations of dopamine in the CNS.
- In addition, HPCB body burden correlated negatively to the free T3 hormone concentrations cross-sectionally as well as longitudinally but not to other thyroid parameters.
- The PCB body burden was negatively correlated with neuropsychological functions (letter fluency) and fine motor performance (steadiness and line tracking accuracy).
- Associations of PCB exposure with liver transaminase activities or with liver structure abnormalities could not be established.
- An elevated odds ratio for diabetes mellitus was found for the third quartile of PCB 52 concentrations at the first examination time point as well as an influence of several PCB congeners on the HbA1c values at the third and fourth examination time point.
- Occurrence of skin hyperpigmentation was correlated to PCB exposure of the participants.

Due to the limited sample size of the HELPcB cohort, further studies in PCB-exposed subjects would be needed to confirm the results summarized here and to establish a potential causal relationship between PCB exposure and clinical effects in humans.
